# The Association between Lipoprotein(a) and Coronary Artery Calcification in Elderly Patients with Diabetes: A Cross-Sectional Study

**DOI:** 10.31083/RCM26114

**Published:** 2025-03-20

**Authors:** Lijun Qiu, Hongwei Qiao

**Affiliations:** ^1^Department of Radiology, Kongjiang Hospital of Yangpu District, 200093 Shanghai, China; ^2^Department of Geriatrics, Kongjiang Hospital of Yangpu District, 200093 Shanghai, China

**Keywords:** lipoprotein(a), coronary artery calcification, diabetes, elderly, restricted cubic spline

## Abstract

**Background:**

Lipoprotein(a) [Lp(a)] is associated with the development of coronary artery calcification (CAC), yet its exact function is not fully understood. This study sought to assess the relationship between Lp(a) levels and the risk of CAC in elderly diabetic patients.

**Methods:**

This cross-sectional study included 486 elderly diabetic patients. The exposure factor was Lp(a) levels, categorized into three groups (T1, T2, T3). The outcome was the presence of CAC. The relationship between Lp(a) levels and CAC was evaluated using several statistical methods, including univariate and multivariate logistic regression, multivariable stratified analysis, receiver operating characteristic (ROC) curve analysis, and restricted cubic spline (RCS) analysis.

**Results:**

The highest Lp(a) group (T3) showed significantly higher prevalence of CAC compared to the T1 and T2 groups. Univariate logistic regression indicated a significant link between Lp(a) and CAC. Furthermore, multivariate logistic regression supported the finding that elevated Lp(a) levels correlated with a heightened risk of CAC in all models. Specifically, each unit rise in Lp(a) was associated with a notable increase in CAC risk, and Log_10_Lp(a) and each 1 standard deviation increase in Lp(a) also significantly elevated CAC risk. Multivariable stratified analysis demonstrated significant differences in CAC risk across various subgroups, including age ≤70 years, males, females, smokers, hypertensive, non-hypertensive, hyperlipidemic, non-hyperlipidemic, non-stroke, and non-chronic kidney disease patients. ROC curve analysis showed that adding Lp(a) to the baseline model improved the area under the curve from 0.741 to 0.755. RCS analysis indicated a significant, approximately linear association between Log_10_Lp(a) and CAC risk (*p* nonlinear = 0.115).

**Conclusions:**

In an elderly diabetic population, elevated levels of Lp(a) were strongly linked to a greater risk of CAC. Integrating Lp(a) measurements with conventional risk factors improves the predictive accuracy for CAC.

## 1. Introduction

Coronary artery disease (CAD) ranks among the top causes of mortality and health-related challenges globally [1]. Atherosclerosis is the underlying pathological process of CAD, with coronary artery calcification (CAC) being an important marker and predictor [2]. The presence of CAC indicates the maturity and calcification of coronary plaques, often signifying a high risk of future cardiovascular events [3]. Among the numerous factors influencing the development of calcification, lipoprotein(a) [Lp(a)] has received increased attention for its potential role in atherosclerosis [4, 5, 6].

Diabetes significantly increases the risk of developing cardiovascular disease (CVD) [7]. Persistent high blood sugar results in various metabolic disruptions, such as dyslipidemia, oxidative stress, and ongoing low-grade inflammation, which all play a role in the onset and advancement of atherosclerosis [8]. Diabetic patients exhibit accelerated atherosclerosis, with plaques that are more extensive and prone to calcification compared to non-diabetic individuals [9]. Elderly diabetic patients are particularly vulnerable due to the combined effects of aging and prolonged exposure to glucose during hyperglycemia, which significantly increases their cardiovascular risk. Aging is linked to issues like endothelial dysfunction, increased arterial stiffness, and heightened systemic inflammation, all of which exacerbate the atherosclerotic process [10]. As a result, older adults with diabetes face a significantly greater risk of developing CAD and suffering from negative cardiovascular events [11].

Lp(a) is a distinct lipoprotein made up of low-density lipoprotein (LDL) and apolipoprotein(a) [apo(a)], which are connected by a disulfide bond. The apo(a) structure exhibits considerable polymorphisms, with variations in the number of kringle IV type 2 repeats among individuals, resulting in notable differences in Lp(a) levels [12]. Research indicates that high Lp(a) levels serve as an independent risk factor for atherosclerotic cardiovascular disease (ASCVD) [13]. Lp(a) promotes atherosclerosis through various mechanisms, including lipid deposition, inflammatory responses, and thrombosis [14]. Due to its structural resemblance to plasminogen, Lp(a) can compete for binding sites within the fibrinolytic system, thereby hindering fibrinolysis and raising the likelihood of thrombosis [14].

Although the relationship between Lp(a) and atherosclerosis has been extensively studied, its specific association with CAC, particularly in elderly diabetic patients, requires further investigation [5]. Elevated Lp(a) levels may promote the development of CAC through multiple mechanisms, including the promotion of smooth muscle cell proliferation by oxidatively modified LDL, inhibition of fibrinolysis, and its pro-inflammatory and pro-thrombotic properties [15]. Current research demonstrates a positive relationship between increased Lp(a) levels and CAC, but the results are inconsistent, possibly due to differences in study populations, methodologies, and confounding factors [16, 17].

Consequently, investigating the link between Lp(a) and CAC in older adults with diabetes holds considerable clinical relevance and practical significance. We hypothesize that higher levels of Lp(a) are significantly associated with an increased risk of CAC in elderly diabetic patients, even after adjusting for other cardiovascular risk factors. First, this population has a markedly increased risk of cardiovascular events. Understanding the role of Lp(a) in the development of CAC can aid in more precise risk stratification and individualized treatment strategies. Second, as a potentially modifiable risk factor, new therapies aimed at lowering Lp(a) levels are under development. Identifying which patients are most likely to benefit will help optimize treatment regimens and improve patient outcomes. Furthermore, elderly diabetic patients often have multiple chronic conditions, making medication management complex. Understanding the role of Lp(a) in this context can provide a basis for developing more effective comprehensive treatment plans, lessening the impact of CVD and enhancing the quality of life for patients.

## 2. Materials and Methods

### 2.1 Research Subjects

This cross-sectional study included 486 elderly diabetic patients who were hospitalized at Kongjiang Hospital of Yangpu District from January 2021 to January 2024. All participants gave informed consent, and the research received approval from the ethics committee at Kongjiang Hospital in Yangpu District (LL-2020-KY-25). The study was conducted in accordance with the Declaration of Helsinki. Inclusion criteria encompassed individuals aged 60 and above who were diagnosed with diabetes mellitus based on the standards set by the American Diabetes Association (ADA) [18]. Exclusion criteria included presence of severe liver disease (such as acute hepatitis, cirrhosis, and liver failure), active malignancy, recent major surgery (within the last six months), severe mental illness, and any other conditions that could affect Lp(a) levels, diabetes, or CAC, such as acute inflammatory diseases, severe infections, and autoimmune disorders.

### 2.2 Data Acquisition and Terminology

Data were collected through patient interviews, medical record reviews, and physical examinations to ensure comprehensiveness and accuracy. Information collected included demographic data, behavioral information, medical history, medication use, and physical measurements. Smoking status was classified into two groups: current smokers and those who had never smoked Medical history included hypertension, hyperlipidemia, stroke, and chronic kidney disease (CKD). Hypertension was identified through a documented diagnosis in medical records, the use of antihypertensive drugs, or by measuring a systolic blood pressure (SBP) of 140 mmHg or higher, or a diastolic blood pressure (DBP) of 90 mmHg or more after five minutes of resting [19]. Hyperlipidemia was identified through documented diagnoses, the use of lipid-lowering medications, or by total cholesterol levels of ≥5.2 mmol/L, LDL cholesterol (LDL-C) of ≥3.4 mmol/L, high-density lipoprotein cholesterol (HDL-C) of <1.0 mmol/L for men or <1.3 mmol/L for women, or triglyceride levels of ≥1.7 mmol/L [20]. Stroke was determined based on a recorded diagnosis of a previous stroke, confirmed by clinical presentation and imaging evidence. CKD was identified through either a documented diagnosis or an estimated glomerular filtration rate (eGFR) of less than 60 mL/min/1.73 m^2^, calculated using the CKD-Epidemiology Collaboration (CKD-EPI) formula [21]. Medication usage was documented for both antihypertensive and lipid-lowering medications. Physical measurements comprised weight and height, which were utilized to compute body mass index (BMI) using the formula weight (kg) divided by height (m) squared.

Laboratory assessments comprised total cholesterol, LDL-C, HDL-C, triglycerides, apolipoprotein A1 (ApoA1), apolipoprotein B (ApoB), fasting plasma glucose (FPG), hemoglobin A1c (HbA1c), and uric acid concentrations All blood biomarkers were measured in fasting patients, from venous blood samples. The eGFR was calculated using the CKD-EPI formula [22]. Blood pressure measurements were taken using a standard method to measure SBP and DBP, with patients resting for at least 5 minutes before each measurement. Each participant underwent three blood pressure measurements, with the average value utilized for the analysis.

### 2.3 Lp(a) Detection and Classification Methods

Lp(a) levels were assessed through an immunoturbidimetric method (Siemens Healthineers, ADVIA Chemistry Lp(a) Assay Kit, Erlangen, Germany). Fasting venous blood samples were collected from patients, centrifuged to separate the serum, and analyzed using a commercial immunoturbidimetric kit containing specific anti-Lp(a) antibodies. The concentration of Lp(a) was determined through turbidimetric measurement. Each assay was calibrated with standard samples and included high, medium, and low concentration quality control samples to ensure accuracy and consistency. Lp(a) levels were expressed in milligrams per liter (mg/L). The general reference range was 0–300 mg/L, although the specific reference range may vary depending on racial and individual differences. Participants were categorized into three groups according to the tertiles of Lp(a) levels: T1 (lowest tertile: ≤154 mg/L), T2 (middle tertile: 154–322 mg/L), and T3 (highest tertile: >322 mg/L).

### 2.4 Detection and Assessment of CAC

CAC was detected using coronary computed tomography angiography (coronary CTA, SOMATOM Definition Flash, Siemens, Berlin, Germany). During the procedure, an iodine contrast agent was injected intravenously to enhance the visualization of the coronary arteries. Patients lay flat on the scanning table with their arms raised above their heads. The computed tomography (CT) scanner captured cross-sectional images of the heart through continuous scanning. To minimize motion artifacts and improve image quality, patients were required to hold their breath during the scan. The scanned images were processed and reconstructed using specialized software to identify and quantify calcified plaques in the coronary arteries. The quantification of CAC was performed using the Agatston scoring system, which calculates the score based on the area and density of the calcified plaques [23]. Image analysis was conducted using specialized software to identify calcified plaques in the coronary arteries. The Agatston score for each plaque was determined by multiplying its area by a density factor derived from its characteristics in the CTA images. The total Agatston score was obtained by summing the scores of all the plaques. Participants were divided into two categories according to their Agatston scores: a non-calcification group (CAC = 0) and a calcification group (CAC >0). This approach enabled precise identification and evaluation of coronary artery calcification, aiding in the subsequent analysis of the relationship between CAC and other factors.

### 2.5 Statistical Analysis

All statistical analyses were performed using relevant software, specifically SPSS version 26.0 (IBM Corp., Armonk, NY, USA) and R version 4.1.3 (R Foundation for Statistical Computing, Vienna, Austria). A two-tailed *p*-value of less than 0.05 was deemed statistically significant. Baseline characteristics were compared among groups with different Lp(a) levels using analysis of variance (ANOVA) for continuous variables and chi-square tests for categorical variables. Univariate logistic regression analysis was performed to evaluate the association between individual variables and CAC, calculating odds ratios (OR) and 95% confidence interval (CI). Multivariate logistic regression analysis was performed using three different models: Model 1 (unadjusted), Model 2 (adjusted for age and gender), and Model 3 (adjusted for age, gender, smoking status, hypertension, CKD, antihypertensive medications, systolic blood pressure, and eGFR) to assess the relationship between Lp(a) levels and CAC while controlling for potential confounding factors. Multivariable stratified analysis was performed to explore potential effect modifications of different subgroups, evaluating the relationship between Lp(a) and CAC across subgroups categorized by age, gender, and smoking status, hypertension, hyperlipidemia, stroke, and CKD. Adjustments were made for age, sex, smoking, hypertension, CKD, antihypertensive drugs, SBP, and eGFR, with interaction terms included in the models to test for statistical interaction. Receiver operating characteristic (ROC) curve analysis was utilized to assess the predictive capability of Lp(a) for CAC, determining the area under the curve (AUC) for Lp(a) by itself, the baseline model (which included age, sex, smoking status, hypertension, CKD, antihypertensive medications, SBP, and eGFR), and the combined model incorporating both Lp(a) and the baseline factors. Comparisons of AUCs determined the added predictive value of Lp(a). Finally, RCS analysis explored the possible nonlinear relationship between Log_10_Lp(a) levels and the risk of CAC. The spline function allowed flexible modeling of the relationship without assuming linearity. The overall significance of the association was assessed, reporting the *p* value for nonlinearity. A *p* value below 0.05 was considered to reflect a significant overall association, while a *p* value of less than 0.05 for nonlinearity indicated a significant nonlinear relationship.

## 3. Results

### 3.1 Baseline Characteristics

Significant differences in baseline characteristics (Table 1) were found among the groups categorized by varying Lp(a) levels. There were significant differences among the three groups T1, T2, and T3 in hypertension, CKD, total cholesterol, LDL-C, ApoA1, ApoB, eGFR, uric acid, and CAC (*p* < 0.05). Specifically, the T3 group exhibited a higher prevalence of CKD, and CAC, along with increased levels of total cholesterol, LDL-C, ApoB, and uric acid, while showing reduced ApoA1 and eGFR levels in comparison to the T1 and T2 groups.

**Table 1.** **Baseline characteristics stratified by Lp(a)**.

		Total patients	T1	T2	T3	*p* value
N		486	164	160	162	
Age, years		72.66 ± 6.65	72.45 ± 7.04	72.42 ± 6.90	73.12 ± 5.99	0.562
Male, n (%)		295 (60.70%)	105 (64.00%)	94 (58.80%)	96 (59.30%)	0.561
Smoking, n (%)		174 (35.80%)	65 (39.60%)	53 (33.10%)	56 (34.60%)	0.437
Medical history, n (%)						
	Hypertension	386 (79.40%)	139 (84.80%)	114 (71.30%)	133 (82.10%)	0.006
	Hyperlipidemia	213 (43.80%)	71 (43.30%)	64 (40.00%)	78 (48.10%)	0.333
	Stroke	143 (29.40%)	48 (29.30%)	46 (28.80%)	49 (30.20%)	0.956
	CKD	157 (32.30%)	39 (23.80%)	54 (33.80%)	64 (39.50%)	0.009
	Antihypertensive drugs	333 (68.50%)	117 (71.30%)	99 (61.90%)	117 (72.20%)	0.086
	Lipid-lowering drugs	11 (2.30%)	5 (3.0%)	4 (2.50%)	2 (1.20%)	0.529
BMI, kg/m^2^		24.66 ± 3.43	24.47 ± 3.35	25.01 ± 3.39	24.51 ± 3.55	0.302
SBP, mmHg		134.63 ± 23.32	133.57 ± 23.93	135.72 ± 23.76	134.64 ± 22.32	0.711
DBP, mmHg		75.80 ± 13.76	76.51 ± 13.61	75.89 ± 14.17	74.99 ± 13.53	0.604
Triglyceride, mmol/L		1.41 (1.04, 2.04)	1.42 (1.04, 2.09)	1.43 (1.07, 1.97)	1.41 (1.02, 2.11)	0.762
Total cholesterol, mmol/L		4.49 ± 1.26	4.29 ± 1.16	4.41 ± 1.21	4.79 ± 1.36	0.001
LDL-C, mmol/L		2.70 ± 0.93	2.48 ± 0.80	2.67 ± 0.88	2.96 ± 1.03	<0.001
HDL-C, mmol/L		1.15 ± 0.28	1.17 ± 0.30	1.13 ± 0.28	1.15 ± 0.27	0.474
ApoA1, g/L		1.06 ± 0.26	1.10 ± 0.27	1.04 ± 0.28	1.03 ± 0.24	0.021
ApoB, g/L		0.84 ± 0.27	0.77 ± 0.22	0.82 ± 0.25	0.92 ± 0.31	<0.001
eGFR, mL/min/1.73 m^2^		74.45 ± 33.25	82.09 ± 36.57	73.10 ± 33.00	68.03 ± 28.27	0.001
Uric acid, µmol/L		362.16 ± 118.75	348.38 ± 117.47	355.16 ± 114.13	383.04 ± 122.32	0.020
FPG, mmol/L		8.30 (7.13, 10.75)	8.38 (7.33, 10.88)	8.42 (7.20, 11.20)	8.01 (7.02, 10.59)	0.453
HbA1c, %		7.80 ± 1.75	7.70 ± 1.65	7.83 ± 1.82	7.86 ± 1.80	0.758
CAC, n (%)						<0.001
	Yes	266 (54.70%)	81 (49.40%)	75 (46.90%)	110 (67.90%)	
	No	220 (45.30%)	83 (50.60%)	85 (53.10%)	52 (32.10%)	

CKD, chronic kidney disease; BMI, body mass index; SBP, systolic blood pressure; DBP, diastolic blood pressure; LDL-C, low-density lipoprotein cholesterol; HDL-C, high-density lipoprotein cholesterol; ApoA1, apolipoprotein A1; ApoB, apolipoprotein B; eGFR, estimated glomerular filtration rate; FPG, fasting plasma glucose; HbA1c, hemoglobin A1c; CAC, coronary artery calcification; Lp(a), lipoprotein(a).

### 3.2 Univariate Logistic Regression Analysis

Univariate logistic regression of Table 2 showed, age (odd ratio (OR) = 1.144, 95% CI: 1.108–1.182, *p* < 0.001), smoking (OR = 1.613, 95% CI: 1.110–2.343, *p* = 0.012), hypertension (OR = 2.011, 95% CI: 1.286–3.144, *p* = 0.002), CKD (OR = 1.656, 95% CI: 1.122–2.444, *p* = 0.011), use of antihypertensive drugs (OR = 2.144, 95% CI: 1.453–3.165, *p* < 0.001), SBP (OR = 1.010, 95% CI: 1.002–1.018, *p* = 0.017), Lp(a) (OR = 1.002, 95% CI: 1.001–1.002, *p* < 0.001), and lower eGFR (OR = 0.992, 95% CI: 0.987–0.998, *p* = 0.006) were all significantly associated with CAC (*p* < 0.05). Conversely, being male (OR = 0.433, 95% CI: 0.296–0.632, *p* < 0.001) was associated with a lower risk of CAC.

**Table 2.** **Univariate logistic regression analysis for CAC**.

	OR (95% CI)	*p* value
Age	1.144 (1.108, 1.182)	<0.001
Male	0.433 (0.296, 0.632)	<0.001
Smoking	1.613 (1.110, 2.343)	0.012
Hypertension	2.011 (1.286, 3.144)	0.002
Hyperlipidemia	1.376 (0.958, 1.976)	0.084
Stroke	1.116 (0.753, 1.653)	0.585
CKD	1.656 (1.122, 2.444)	0.011
Antihypertensive drugs	2.144 (1.453, 3.165)	<0.001
Lipid-lowering drugs	3.817 (0.816, 17.855)	0.089
BMI	0.960 (0.911, 1.011)	0.123
SBP	1.010 (1.002, 1.018)	0.017
DBP	0.996 (0.983, 1.009)	0.508
Triglyceride	0.953 (0.818, 1.110)	0.535
Total cholesterol	1.113 (0.964, 1.286)	0.143
LDL-C	1.131 (0.931, 1.374)	0.215
HDL-C	1.606 (0.842, 3.064)	0.150
ApoA1	1.283 (0.648, 2.541)	0.474
ApoB	1.591 (0.806, 3.140)	0.181
Lp(a)	1.002 (1.001, 1.002)	<0.001
eGFR	0.992 (0.987, 0.998)	0.006
Uric acid	1.001 (0.999, 1.003)	0.205
FPG	0.970 (0.933, 1.008)	0.123
HbA1c	0.987 (0.874, 1.116)	0.837

CAC, coronary artery calcification; CKD, chronic kidney disease; BMI, body mass index; SBP, systolic blood pressure; DBP, diastolic blood pressure; LDL-C, low-density lipoprotein cholesterol; HDL-C, high-density lipoprotein cholesterol; ApoA1, apolipoprotein A1; ApoB, apolipoprotein B; Lp(a), lipoprotein(a); eGFR, estimated glomerular filtration rate; FPG, fasting plasma glucose; HbA1c, hemoglobin A1c; OR, odd ratio; CI, confidence interval.

### 3.3 Multivariate Logistic Regression Analysis

In the multi-model analysis examining the relationship between Lp(a) and CAC (Table 3), Model 1 (unadjusted), Model 2 (adjusted for age and gender), and Model 3 (adjusted for age, gender, smoking status, hypertension, CKD, antihypertensive medications, SBP, and eGFR) all produced significant findings. Specifically, in comparison to the T1 group, the T3 group exhibited an increased risk of CAC across all models (Model 1: OR = 2.168, 95% CI: 1.382–3.400, *p* = 0.001; Model 2: OR = 2.164, 95% CI: 1.326–3.532, *p* = 0.002; Model 3: OR = 2.210, 95% CI: 1.347–3.625, *p* = 0.002). In all models, each unit rise in Lp(a) corresponded to a significant increase in the risk of CAC (Model 1: OR = 1.002, 95% CI: 1.001–1.002, *p* < 0.001; Model 2: OR = 1.002, 95% CI: 1.001–1.002, *p* = 0.001; Model 3: OR = 1.002, 95% CI: 1.001–1.003, *p* = 0.001). Log_10_Lp(a) also significantly increased the risk of CAC in all models (Model 1: OR = 2.408, 95% CI: 1.524–3.806, *p* < 0.001; Model 2: OR = 2.239, 95% CI: 1.352–3.705, *p* = 0.002; Model 3: OR = 2.344, 95% CI: 1.405–3.911, *p* = 0.001). Similarly, in all models, a 1 standard deviation rise in Lp(a) corresponded to a notable increase in the risk of CAC (Model 1: OR = 1.472, 95% CI: 1.200–1.804, *p* < 0.001; Model 2: OR = 1.466, 95% CI: 1.177–1.826, *p* = 0.001; Model 3: OR = 1.483, 95% CI: 1.186–1.854, *p* = 0.001). Furthermore, the *p*-values for the trend analysis were all below 0.001, demonstrating a significant elevation in CAC risk associated with higher Lp(a) levels.

**Table 3.** **Association of Lp(a) with CAC**.

	Model 1		Model 2		Model 3	
	OR (95% CI)	*p* value	OR (95% CI)	*p* value	OR (95% CI)	*p* value
Lp(a): T1	Ref	-	Ref	-	Ref	-
Lp(a): T2	0.904 (0.585, 1.398)	0.651	0.849 (0.523, 1.379)	0.509	0.908 (0.556, 1.483)	0.699
Lp(a): T3	2.168 (1.382, 3.400)	0.001	2.164 (1.326, 3.532)	0.002	2.210 (1.347, 3.625)	0.002
*p* for trend		<0.001		<0.001		0.001
Lp(a) (per 1-unit)	1.002 (1.001, 1.002)	<0.001	1.002 (1.001, 1.002)	0.001	1.002 (1.001, 1.003)	0.001
Log_10_Lp(a)	2.408 (1.524, 3.806)	<0.001	2.239 (1.352, 3.705)	0.002	2.344 (1.405, 3.911)	0.001
Lp(a) (per 1 SD)	1.472 (1.200, 1.804)	<0.001	1.466 (1.177, 1.826)	0.001	1.483 (1.186, 1.854)	0.001

Model 1: unadjusted; Model 2: adjusted for age and sex; Model 3: adjusted for age, sex, smoking, hypertension, CKD, antihypertensive drugs, systolic blood pressure, and eGFR. Lp(a), lipoprotein(a); CAC, coronary artery calcification; CKD, chronic kidney disease; eGFR, estimated glomerular filtration rate; OR, odd ratio; CI, confidence interval; Ref, reference.

### 3.4 Multivariable Stratified Analysis

In the multivariable stratified analysis (Table 4), adjustments were made for age, sex, smoking status, hypertension, CKD, antihypertensive drugs, SBP, and eGFR. The association between Lp(a) and CAC showed significant differences across multiple subgroups. Specifically, the T3 group exhibited significantly higher CAC risk in subgroups including age ≤70 years, males, females, smokers, hypertensive patients, non-hypertensive patients, hyperlipidemic patients, non-hyperlipidemic patients, non-stroke patients, and non-CKD patients (*p* < 0.05).

**Table 4.** **Multivariable stratified association between Lp(a) and CAC**.

		Lp(a): T1	Lp(a): T2	Lp(a): T3	*p* trend	*p* interaction
		OR (95% CI)	OR (95% CI)	OR (95% CI)		
Age						0.122
	≤70 years	Ref	1.006 (0.342, 2.957)	4.513 (1.653, 12.319)**	0.004	
	>70 years	Ref	0.895 (0.473, 1.696)	1.366 (0.716, 2.605)	0.413	
Sex						0.872
	Male	Ref	0.731 (0.380, 1.406)	2.023 (1.071, 3.825)*	0.008	
	Female	Ref	1.056 (0.490, 2.274)	2.357 (1.019, 5.451)*	0.080	
Smoking						0.245
	Yes	Ref	1.047 (0.383, 2.859)	3.044 (1.185, 7.821)*	0.033	
	No	Ref	0.774 (0.428, 1.400)	1.834 (0.996, 3.380)	0.016	
Hypertension						0.195
	Yes	Ref	0.763 (0.437, 1.333)	1.811 (1.054, 3.114)*	0.009	
	No	Ref	1.984 (0.573, 6.867)	6.769 (1.768, 25.918)**	0.012	
Hyperlipidemia						0.150
	Yes	Ref	1.250 (0.565, 2.765)	2.185 (1.004, 4.755)*	0.126	
	No	Ref	0.792 (0.418, 1.503)	2.209 (1.133, 4.309)*	0.008	
Stroke						0.660
	Yes	Ref	0.676 (0.272, 1.682)	1.560 (0.642, 3.789)	0.185	
	No	Ref	1.023 (0.560, 1.871)	2.625 (1.421, 4.849)**	0.002	
CKD						0.469
	Yes	Ref	1.374 (0.511, 3.691)	2.588 (0.985, 6.798)	0.123	
	No	Ref	0.675 (0.378, 1.207)	2.009 (1.111, 3.633)*	0.002	

*p < 0.05, **p < 0.01. Lp(a), lipoprotein(a); CAC, coronary artery calcification; CKD, chronic kidney disease; OR, odd ratio; CI, confidence interval; Ref, reference.

### 3.5 ROC Curve Analysis

**Fig. 1.**
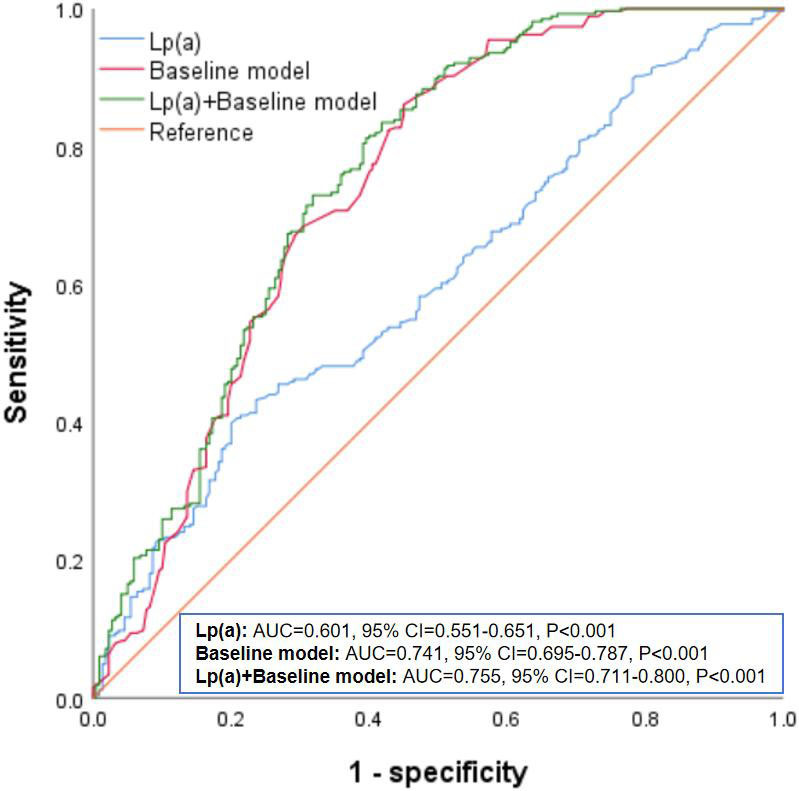
**ROC analysis of Lp(a) in predicting CAC**. Lp(a), lipoprotein(a); CAC, coronary artery calcification; ROC, receiver operating characteristic; AUC, area under the curve; CI, confidence interval.

Fig. 1 showed the ROC curve analysis results of Lp(a) in predicting CAC. The baseline model consisted of all variables from Table 3 (age, sex, smoking, hypertension, CKD, antihypertensive drugs, SBP, and eGFR). The results indicated that the AUC for predicting CAC using Lp(a) alone was 0.601 (95% CI: 0.551–0.651, *p* < 0.001). The AUC for the baseline model was 0.741 (95% CI: 0.695–0.787, *p* < 0.001). When Lp(a) was added to the baseline model, the AUC increased to 0.755 (95% CI: 0.711–0.800, *p* < 0.001).

### 3.6 RCS Analysis

**Fig. 2.**
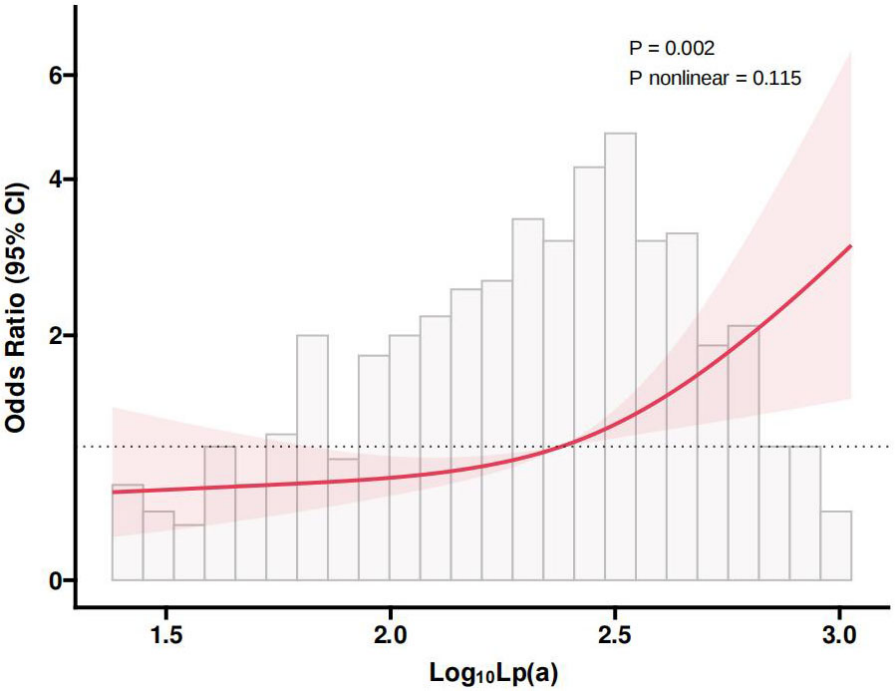
**RCS plot of the nonlinear association between Lp(a) and CAC**. Lp(a), lipoprotein(a); CAC, coronary artery calcification; RCS, restricted cubic spline; CI, confidence interval.

Fig. 2 presented the RCS plot illustrating the nonlinear relationship between Lp(a) and CAC. The findings revealed a significant link between Log_10_Lp(a) and CAC risk (*p* = 0.002), while the *p*-value for nonlinearity was 0.115, indicating it was not statistically significant. This implied that as the Log_10_Lp(a) increased, the risk of CAC also significantly increased, and this relationship was approximately linear within the observed range.

## 4. Discussion

This study, conducted a cross-sectional analysis of 486 elderly diabetic patients, found a significant positive association between Lp(a) levels and the risk of CAC. The group with the highest Lp(a) levels (T3) demonstrated a notably greater prevalence of CAC, a result supported by both univariate and multivariate logistic regression analyses. Additionally, the multivariate logistic regression confirmed that elevated Lp(a) levels correlated with a higher risk of CAC, with each unit increase in Lp(a) significantly raising this risk. Stratified analysis indicated that the impact of Lp(a) levels on CAC risk was consistent across different subgroups. The ROC curve analysis demonstrated that adding Lp(a) levels to the baseline model improved the AUC from 0.741 to 0.755, indicating enhanced predictive accuracy for CAC. The RCS analysis revealed a notable linear association between Log_10_Lp(a) and the risk of CAC.

Currently, while numerous studies have identified a link between Lp(a) and CVD, including its severity, adverse outcomes, and mortality, the connection between Lp(a) and CAC remains inconsistent [24, 25, 26, 27, 28]. Consistent with our study, certain studies have indicated a positive association between increased Lp(a) levels and CAC. For instance, Ong *et al*. [29] discovered that increased plasma Lp(a) levels were significantly linked to a rise in CAC volume, particularly in those with elevated inflammatory and coagulation markers, which suggests that Lp(a) could act as a possible biomarker for the progression of CAC volume, highlighting its importance in cardiovascular disease risk assessment. Additionally, a substantial prospective cohort study from the Multi-Ethnic Study of Atherosclerosis (MESA) found that increased Lp(a) levels were linked to a faster progression of CAC [30]. A meta-analysis involving 23,105 asymptomatic patients conducted by Martignoni *et al*. [16] discovered a significant link between increased Lp(a) levels and an elevated risk of CAC, particularly with a CAC >0 and CAC ≥100. The study underscores the potential of Lp(a) as an atherogenic factor, aiding in the advancement of coronary calcification as time passes. Additionally, Vazirian *et al*. [31] performed a systematic review and meta-analysis that incorporated 12 studies, comprising 8 cross-sectional studies with 18,668 participants and 4 cohort studies with 15,355 participants. Their findings indicated a significant association between elevated Lp(a) levels and CAC in asymptomatic individuals with cardiovascular disease. A recent systematic review and meta-analysis by Qiu *et al*. [32] examined 17 studies involving 40,073 participants to re-evaluate the link between plasma Lp(a) and CAC. Their findings indicated that higher Lp(a) levels were significantly associated with both the occurrence and advancement of CAC, suggesting that monitoring and managing Lp(a) levels may aid in the early identification and prevention of CAC progression, thereby reducing the likelihood of developing CVD. Nonetheless, various other studies failed to validate the independent link between Lp(a) and CAC. For example, Jackson *et al*. [17] utilized data from MESA to analyze 5597 participants who did not have clinical ASCVD and were not using statin medications, to evaluate the relationship between Lp(a) levels and initial CAC volume/density, as well as its progression. The study found that, in comparison to other lipid biomarkers, Lp(a) did not show a significant correlation with baseline CAC volume or density and was only weakly linked to changes in volume over time, indicating that Lp(a) is not as robust a predictor of CAC as other lipid biomarkers. Additionally, Kim *et al*. [33] performed a single-center observational study involving 252 patients who had coronary CTA and plasma Lp(a) assessments, discovering no significant correlation between Lp(a) levels and CAC scores. Guerra *et al*. [34] analyzed the relationship between plasma Lp(a) levels and CAC in a population-based study of 761 black and 527 white participants, and found no considerable connection of Lp(a) levels with CAC in either group. In a study with 765 hypertensive participants, Kullo *et al*. [35] discovered that Lp(a) levels did not show a significant correlation with CAC scores in either gender, and this finding was not altered by the estimated 10-year risk for coronary heart disease. However, these studies did not explore this association in detail within specific populations such as elderly diabetic patients. Unlike these studies, our research not only provided comprehensive evidence supporting Lp(a) as a potential predictor of CAC through multivariate stratified analysis and RCS, but also demonstrated that incorporating Lp(a) levels into the predictive model significantly enhanced its accuracy in elderly diabetic patients.

The mechanisms by which Lp(a) influences atherosclerosis and CAC are multifaceted. First, the structure of Lp(a) contains components similar to plasminogen, allowing it to compete for binding sites in the fibrinolytic system, thereby inhibiting fibrinolysis and increasing the risk of thrombosis. In this manner, Lp(a) can enhance the instability of atherosclerotic plaques and promote the development of CAC [15]. Second, oxidized LDL may promote the proliferation and migration of smooth muscle cells, further enhancing arterial wall calcification. The oxidized LDL component within Lp(a) can activate smooth muscle cells, transforming them into osteoblast-like cells, thereby depositing calcium salts in the arterial wall and promoting CAC formation [14]. Third, Lp(a) possesses pro-inflammatory properties, activating inflammatory cells and releasing pro-inflammatory cytokines, which increase inflammation in the arterial wall. This inflammatory response not only leads to the instability of atherosclerotic plaques, but also promotes the calcification process, thereby increasing the risk of CAC [36]. Fourth, the pro-thrombotic nature of Lp(a) increases the risk of thrombosis within atherosclerotic plaques, further enhancing plaque instability and calcification. By increasing the frequency of thrombus formation within plaques, Lp(a) can accelerate the development of CAC [37].

This study has several limitations. Firstly, being a cross-sectional study, it does not allow for the determination of a causal relationship between Lp(a) levels and CAC. Longitudinal studies in the future are necessary to confirm this association. Secondly, the participants were exclusively elderly diabetic patients, which may limit the generalizability of the findings to other groups, especially non-diabetic or younger individuals. Research in more diverse populations is needed to verify the generalizability of our findings. Additionally, although multiple confounding factors were adjusted for in the statistical analysis, unmeasured confounders may still influence the results. For instance, genetic polymorphisms and lifestyle factors might affect Lp(a) levels and CAC risk. Furthermore, the sample size and regional scope of this study may limit the generalizability of the results. Verification in larger-scale studies and in different regions is necessary to confirm the reliability and broad applicability of these research findings. Finally, one of the limitations of this study was that it did not explore the “zero calcium phenomenon” in CAC and the potential progression of calcification in the future. Previous research has shown that the prevalence of a zero CAC score in the Asian population is 18.2%, and the risk of subclinical CAC progression may increase after five years [38]. Furthermore, differences in subclinical CAC progression between genders were not addressed in this study. Therefore, future research should focus on the zero calcium phenomenon and the long-term progression of CAC to provide a more comprehensive basis for cardiovascular risk assessment in the Asian population.

## 5. Conclusions

In summary, this research demonstrated a significant relationship between elevated Lp(a) levels and a greater risk of CAC among older diabetic patients. Incorporating Lp(a) levels with traditional risk factors improves the predictive accuracy for CAC. Understanding the role of Lp(a) in the development of CAC can aid in more precise risk stratification and individualized treatment strategies, ultimately improving cardiovascular health and overall prognosis in this high risk population. Future studies should aim to be large-scale, prospective research projects to validate the causal link between Lp(a) and CAC, as well as to explore the specific mechanisms through which Lp(a) contributes to CAC. Additionally, exploring interventions to lower Lp(a) levels and their effects on CAC progression and cardiovascular events is crucial. Investigating other potential biomarkers and imaging techniques will also help to enhance the accuracy of CAC risk assessment, providing more effective clinical tools to manage these high risk populations.

## Availability of Data and Materials

The raw data used in this study is available from the corresponding author upon reasonable request.
